# Temporal changes in anthropogenic seismic noise levels associated with economic and leisure activities during the COVID-19 pandemic

**DOI:** 10.1038/s41598-021-00063-6

**Published:** 2021-11-05

**Authors:** Hiro Nimiya, Tatsunori Ikeda, Takeshi Tsuji

**Affiliations:** 1 Geological Survey of Japan, National Institute of Advanced Industrial Science and Technology (AIST), Ibaraki, Japan; 2 Department of Earth Resources Engineering, Kyushu University, Fukuoka, Japan; 3 International Institute for Carbon-Neutral Energy Research (I2CNER), Kyushu University, Fukuoka, Japan; 4 Disaster Prevention Research Institute, Kyoto University, Kyoto, Japan

**Keywords:** Solid Earth sciences, Seismology

## Abstract

Seismic ambient noise with frequencies > 1 Hz includes noise related to human activities. A reduction in seismic noise during the COVID-19 pandemic has been observed worldwide, as restrictions were imposed to control outbreaks of the SARS-CoV-2 virus. In this context, we studied the effect of changes in anthropogenic activities during COVID-19 on the seismic noise levels in the Tokyo metropolitan area, Japan, considering time of day, day of the week, and seasonal changes. The results showed the largest reduction in noise levels during the first state of emergency under most conditions. After the first state of emergency was lifted, the daytime noise reverted to previous levels immediately on weekdays and gradually on Sundays. This was likely because economic activities instantly resumed, while non-essential outings on Sundays were still mostly avoided. Furthermore, the daytime noise level on Sundays was strongly reduced regardless of changes on weekdays after the second state of emergency, which restricted activities mainly at night. Sunday noise levels gradually increased from the middle of the second state of emergency, suggesting a gradual reduction in public concern about COVID-19 following a decrease in the number of infections. Our findings demonstrate that seismic noise can be used to monitor social activities.

## Introduction

Ambient seismic noise recorded by seismometers includes microseisms and anthropogenic or cultural noise. Microseisms are caused by the coupling between the ocean and the solid Earth at frequencies mostly < 1 Hz^1^, whereas anthropogenic seismic noise, especially in urban areas, includes seismic signals generated by human activities such as moving people and industrial activities^2–5^. A recent study reported an increase in ambient seismic noise in response to an increase in the gross domestic product (GDP), which is associated with the magnitude of human activity^6^. Because an increase in ambient noise can be attributed to a wide range of human activities, ambient seismic noise in urban areas tends to be stronger and more complex. For instance, Díaz et al.^5^ monitored road traffic and subway trains along an avenue approximately 150 m away for frequencies > 1 Hz. They succeeded in detecting signals generated by various anthropogenic noises, including not only road traffic but also rock concerts and football games. Considering the temporary absence of a specific noise source, Green et al.^7^ monitored the ambient seismic noise during a complete shutdown of the subway system during an industrial strike and found a clear reduction for frequencies > 25 Hz within a distance of 100 m, but no reduction at a distance of ~ 600 m from the nearest subway line. Hence, ambient seismic noise includes various anthropogenic noises, depending on the frequency, time, and distance^8, 9^.

The outbreak of the SARS-CoV-2 virus responsible for COVID-19 has immensely impacted and restricted the routine lives of people worldwide. After the first report of COVID-19 in December 2019, it spread rapidly and exponentially around the globe, and the World Health Organization (WHO) declared it a pandemic on March 13, 2020^10^. Many countries went into lockdown to contain major COVID-19 outbreaks and daily life changed globally during the COVID-19 pandemic^11^. The large-scale restrictions on human activities during the pandemic also presented a rare opportunity to clearly distinguish anthropogenic seismic noise from ambient seismic noise. Poli et al.^12^ and Xiao et al.^13^ compared ambient seismic noise levels before and during pandemic-related lockdowns by using continuous seismic records for Italy and China, respectively, and observed a clear decrease in noise levels during lockdown. Poli et al.^12^ found that the noise level was significantly reduced in the frequency band of 1–10 Hz. For China, the decrease in noise level was much larger than that for Italy^13^. Xiao et al.^13^ inferred that this was caused by the difference in the strictness of enforcing restrictions by the governments of the two countries. Lecocq et al.^14^ investigated noise levels worldwide. They found that the noise level in the frequency range of 4–14 Hz was sensitive to lockdowns and was reduced by up to 50% during lockdown. While the reduction was highest in populated areas, the seismic quiescence extended for several kilometers. At the local scale, continuous fiber-optic distributed acoustic sensing recordings showed a spatial reduction in noise levels corresponding to COVID-19 responses^15^. A 50% decrease in vehicular noise was observed in one commuter sector immediately following a stay-at-home order, even though the traffic near hospitals persisted.

**Figure 1:**
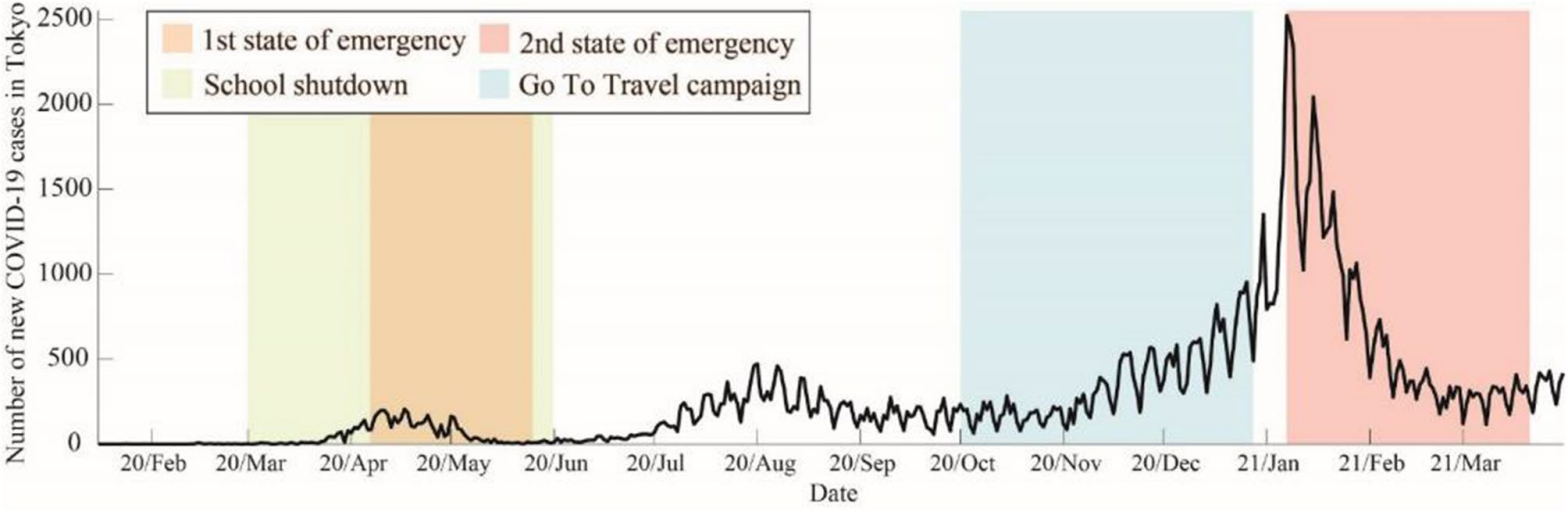
Number of new COVID-19 cases in Tokyo. The black solid line indicates the number of new COVID-19 cases in Tokyo; the orange and pink shaded areas represent the periods of the first and second states of emergency states in Tokyo, respectively; the green shaded area represents the period of school closures; and the light blue shaded area represents the period of the Go To Travel campaign.

Since the first case was reported on January 28, 2020, the number of confirmed cases in Japan increased, with the case curve showing several peaks. Figure 1 shows the number of new COVID-19 cases in Tokyo. The national and local governments took measures against the tide of COVID-19, depending on the number of new cases. The first wave started in late February 2020. To prevent the spread of infection, restrictions, such as school closures and the cancellation of public events, were issued. In Tokyo, schools remained closed from March 2 to June 30, 2020 (yellow shaded area in Fig. 1). In response to the COVID-19 pandemic, the Japanese government declared the first state of emergency in Tokyo from April 7 to May 25, 2020 (pink shaded area in Fig. 1), during which many people voluntarily refrained from going out. However, trains and road traffic did not stop because economic activities continued. After the first wave, the Japanese government took measures to improve the economy, such as encouraging domestic travel. In Tokyo, based on the daily case situation, domestic travel was encouraged from October 2020; the government campaign was called “Go To Travel” (light blue shaded area in Fig. 1). However, these economic recovery measures were stopped on December 28 because the third wave started. Because the number of new COVID-19 cases rapidly increased in the third wave, a second state of emergency was declared from January 7 to March 21, 2021 (purple shaded area in Fig. 1). The second state of emergency focused on restricting activity at night.

Yabe et al.^16^ reported a reduction in seismic noise in the Tokyo metropolitan area associated with reduced social activity due to COVID-19 during the first wave. To monitor the seismic noise level, data from 18 seismic stations of the Metropolitan Seismic Observation Network (MeSO-net) for the period January 2018 to June 2020 were used. They observed a two-step seismic noise reduction. The first reduction occurred when schools were closed at frequencies mainly > 20 Hz. The second reduction occurred when the first state of emergency was declared, mainly in the frequency range of 1–20 Hz, reflecting a decrease in social activities in the entire Tokyo metropolitan area^16^.

In this study, we monitored temporal changes in the seismic noise level around Tokyo one year after the outbreak of COVID-19, using data from 101 seismic stations from April 2017 to mid-March 2021. We investigated the variation in the noise level in different time periods and on different days of the week in the two frequency bands in which the noise reduction was reported previously. In addition, since the estimated temporal variation in the noise level showed clear seasonal variations, we modeled the seasonal variations by assuming a simple annual cycle based on the data of the past 2 years. We then emphasized the temporal variations in the noise level due to COVID-19 by correcting for the seasonal variations.

### Data source and calculation of power spectral density (PSD)

**Figure 2:**
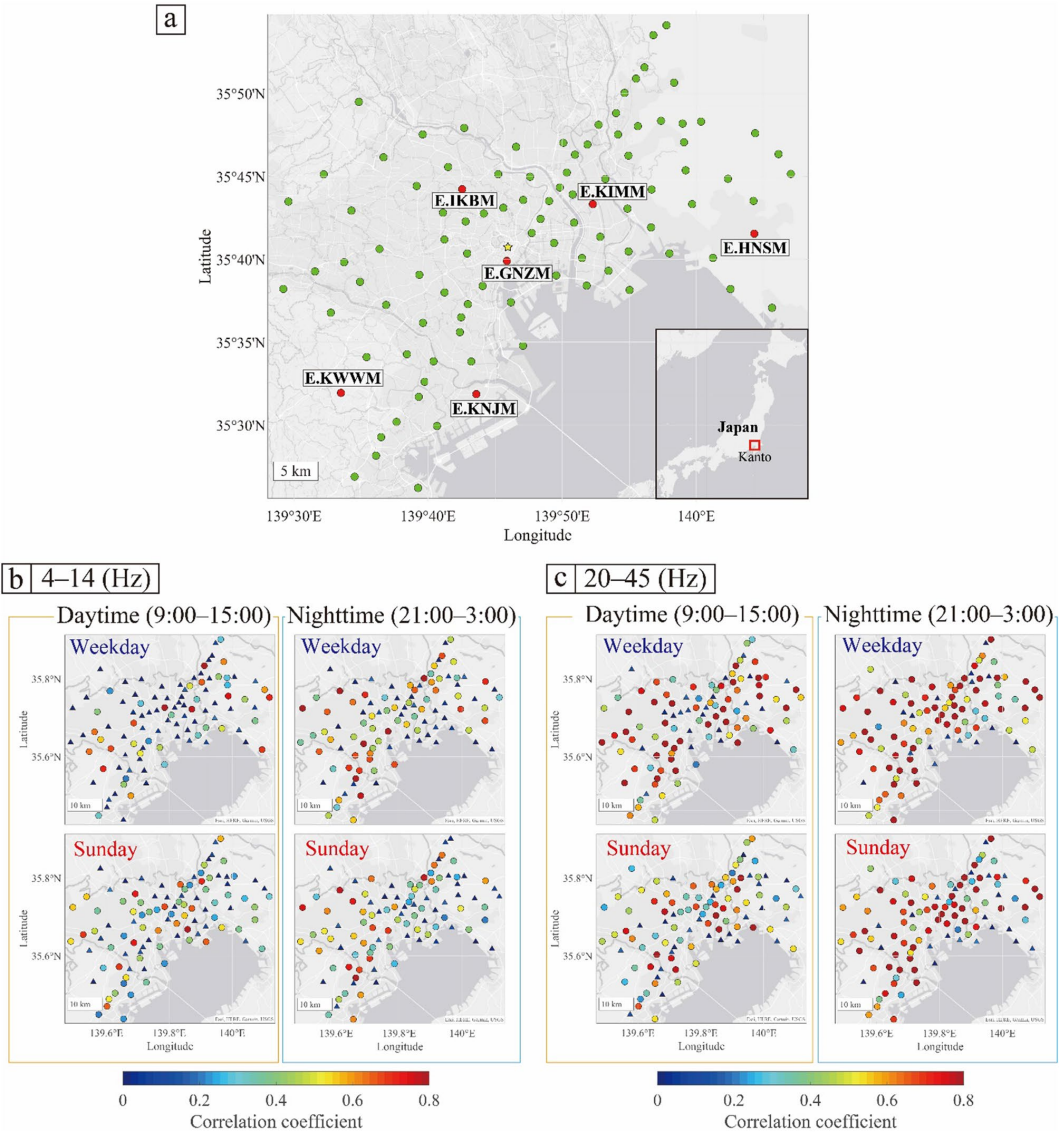
(**a**) Arrangement of the MeSO-net. Red and green dots show the MeSO-net stations. The yellow star indicates the Tokyo station. (**b**, **c**) Map of correlation coefficients, which were calculated considering day and time in the frequency range of (**b**) 4–14 Hz and (**c**) 20–45 Hz. Triangles represent the stations that showed correlation coefficients < 0.2 and circles represent the stations with correlation coefficients > 0.2; the latter were used in Figs. 6, 7, and 8.

We used publicly available, continuous, three-component ground acceleration data recorded by the MeSO-net. The MeSO-net stations have acceleration sensors with a sampling rate of 200 Hz and are installed at depths of ~ 20 m to reduce the influence of anthropogenic noise^17, 18^. Approximately 300 MeSO-net stations are deployed around the metropolitan area, of which we chose 101 stations located around Tokyo (Fig. 2a). The vertical component of the seismic noise from April 1, 2017, to March 15, 2021, was used in this study.

To estimate the temporal change in ambient noise before and after the outbreak of COVID-19, we calculated the power spectral density (PSD) following the method of McNamara and Boaz^19^. The PSD as a function of time and frequency is an effective tool for visually distinguishing different types of seismic noise. We divided the daily data into 10-min segments and applied demean and detrend to each data segment. After applying a 50% cosine taper, we calculated the PSD for each 10-min data segment. The decrease in the amplitude value due to the taper was corrected using the coefficient $c=\sqrt{N/{\sum }_{k}^{N}{w\left(k\right)}^{2}}$, where $N$ is the length of the window of the taper and $w\left(k\right)$ is the window of the taper for the *k*th sample. In this study, we used the median values of the PSDs for the specific periods (e.g., all day, daytime, or night) as the daily PSDs because the mean value is easily affected by large outliers caused by earthquakes, nearby construction, etc.

### Temporal changes in relative noise level

To monitor the PSD, we estimated the temporal changes in the relative noise level ($R$) at each station using the following equation:

1 $$R \left(\text{\%}\right)=\frac{{PSD}_{cur}-{PSD}_{ref}}{{PSD}_{ref}}\times 100,$$

where ${PSD}_{cur}$ represents the daily PSD, which was estimated using the median value of all segments during specific time segments and ${PSD}_{ref}$ represents the median value of 2 years of ${PSD}_{cur}$ from April 2017 to March 2019. The PSDs were used without logarithmic transformation.

**Figure 3:**
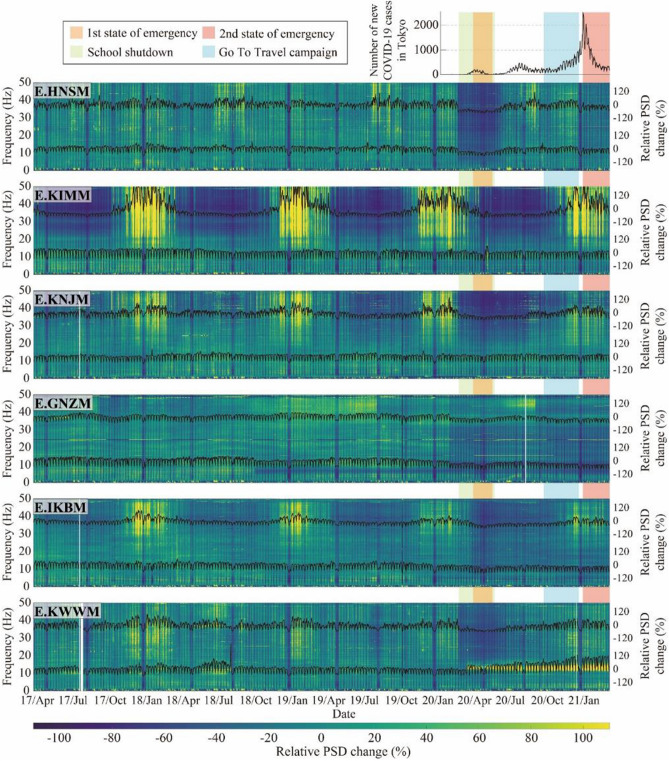
Time-series of the relative noise level from April 2017 to March 2021 for six stations. The background color indicates the relative noise level at each frequency. The upper and lower black solid lines in each panel represent the relative noise level of the mean value in the frequency ranges of 4–14 Hz and 20–45 Hz, respectively. The upper right figure shows the number of new COVID-19 cases in Tokyo (same as Fig. 1). The orange and pink shaded areas represent the periods of the first and second states of emergency in Tokyo, respectively; the green shaded area represents the period of school closures; and the light blue shaded area represents the period of the Go To Travel campaign.

Figure 3 shows the temporal changes in the relative noise level ($R$) at several stations, calculated using the ${PSD}_{cur}$ for all segments in a day. The largest decrease in PSD was observed during the first state of emergency at most stations (e.g., E.HNSM and E.KNJM). This variation was larger at higher frequencies, consistent with the results of a previous study^16^. To clarify the differences in variation with frequency, we focused on the two frequency bands of 4–14 Hz and 20–45 Hz. The high-frequency range of 20–45 Hz was previously found to show a two-step seismic noise reduction^16^, whereas the low-frequency range of 4–14 Hz was found to show a clear reduction during lockdowns worldwide^14^. The mean PSD value was calculated for each frequency band, and the temporal change in the relative noise level was calculated using Eq. (1) (black solid lines in Fig. 3). The variations in the PSD were different in each frequency range. Some stations showed clear seasonal variations, especially in the higher frequency range (e.g., E.KIMM), whereas they were less pronounced at lower frequencies.

### Variation with time of day

The anthropogenic noise levels throughout the day are known to have distinct variations due to variations in human activities with the time of day^2, 3, 14, 16, 20^. In this study, we estimated the temporal variations of the PSD for each time of day to determine which times of day are more sensitive to changes in human activities related to COVID-19. The temporal variations of PSD depending on the time of day was estimated by calculating ${PSD}_{cur}$ for a specific time period. The time range was set to 3 h. To eliminate the effect of the day of the week and calendar effects, we removed bank holidays and the periods around long holidays (New Year holidays: December 26–January 7, summer holidays: August 10–August 17, and consecutive holidays: April 27–May 7) and smoothed the data using a 7-day moving window. Although the temporal variations of PSD showed different trends depending on the station, the median values of the variations of all stations were used to determine the overall trend of the variation.

**Figure 4:**
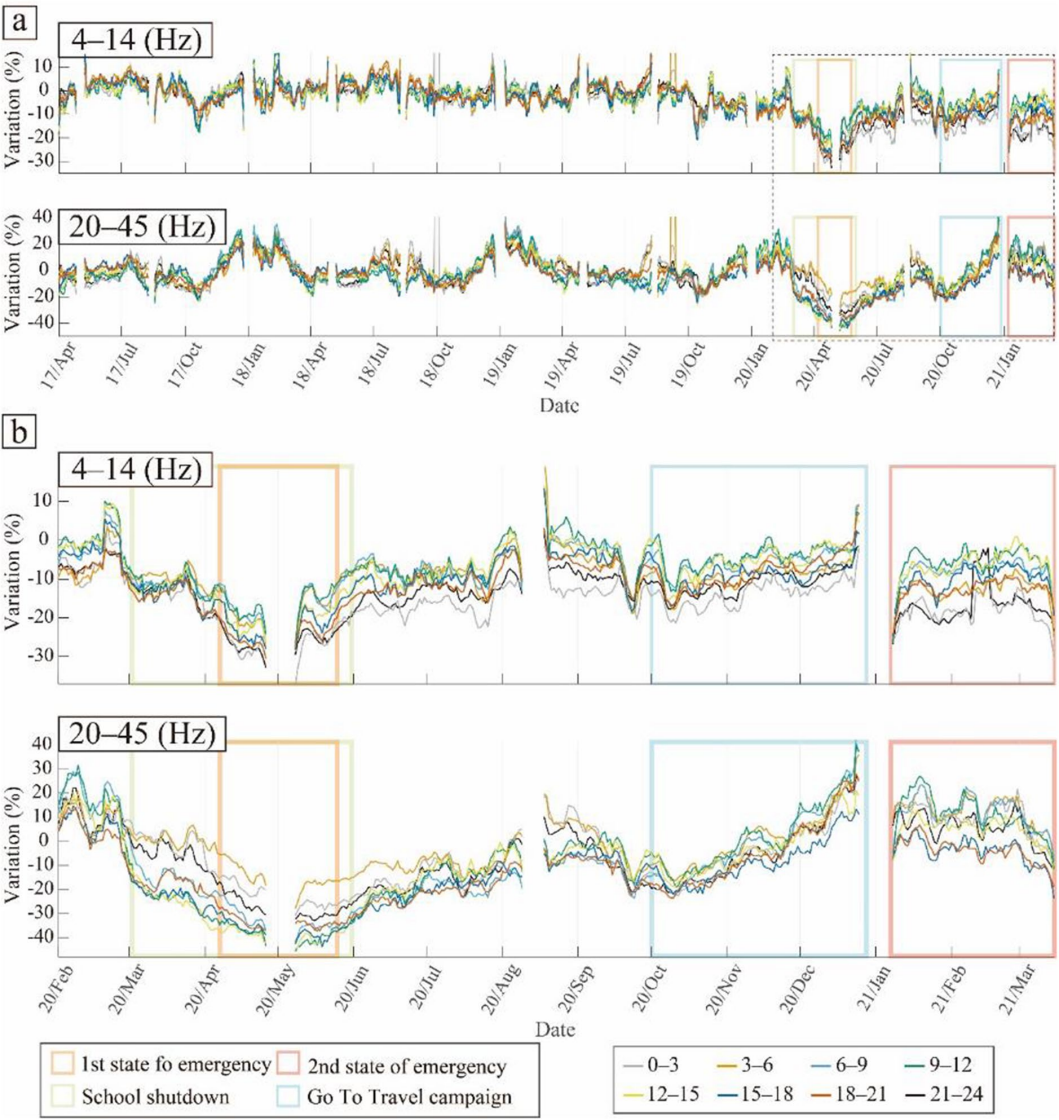
Temporal variations of PSD depending on the time of day. (**a**) From April 2017 to March 2020. (**b**) Enlarged view of the black dashed square in (**a**). Each solid line indicates the PSD in each time period shown in the right bottom box. The orange and pink squares represent the periods of the first and second states of emergency in Tokyo, respectively; the green square represents the period of school closures; and the light blue square represents the period of the Go To Travel campaign.

Figure 4 shows the temporal variation of PSD with time of day and, as can be seen, the overall trend of the PSD variations significantly differs between the two frequency bands. In the high-frequency range (20–45 Hz), the PSD rapidly decreased following the school closures, whereas this trend was not clear in the low-frequency range (4–14 Hz), as discussed in a previous study^16^. The long-period temporal variations of PSD showed strong seasonal variations in the high-frequency range (20–45 Hz), whereas they were not clearly visible in the low-frequency range (4–14 Hz) (Fig. 4a). After the outbreak of COVID-19, the difference in the temporal variations of PSD with time of day became larger in both frequency bands (Fig. 4b).

In the low-frequency range (4–14 Hz), the PSD decreased in all time periods during the first declared state of emergency and quickly recovered to near previous levels after the state of emergency was lifted (Fig. 4). The degree of PSD reduction depended on the time of the day. The largest reductions were observed during the period from 21:00 to 3:00 in most periods. Interestingly, differences in the variations of PSD between daytime and nighttime were still observed after the first state of emergency was lifted.

In the high-frequency range (20–45 Hz), the daytime PSD was clearly reduced during the school closures before the state of emergency was declared (Fig. 4). This reflects a decrease in the noise level due to the absence of school-related activities. Because MeSO-net stations are mainly installed in schools, the PSDs observed at MeSO-net stations are more sensitive to noise related to school activities^16^. However, considering the large PSD reductions from 6:00 to 21:00, these reductions do not only reflect a decrease in school activities but also of other human activities (e.g., traffic). The PSD during the Go To Travel campaign gradually increased in all time periods, and the differences in the time-dependent PSD variations became smaller. During the second declared state of emergency, the PSD decreased significantly from 15:00 to 21:00. This was because the second state of emergency was targeted to restrict nonessential and non-urgent outings, especially at night.

### Variation with day of the week

In the analysis of the variation of the PSD depending on the time of day, the effect of the day of the week was ignored by using a 7-day moving window (Fig. 4). Because the difference in the PSD between weekdays and Sundays was significant (Fig. 3), the differences in the temporal variations of PSD of the day of the week were also examined. In this study, the temporal variations of PSD were calculated separately for weekdays and Sundays. Bank holidays and the periods around long holidays were excluded. For weekdays, the data was smoothed using a 7-day moving window after weekday data extraction. Considering the differences in PSD based on the time of day (Fig. 4), we calculated the temporal variations of PSD during the daytime (9:00–15:00), the evening (15:00–21:00), and at night (21:00–3:00).

**Figure 5:**
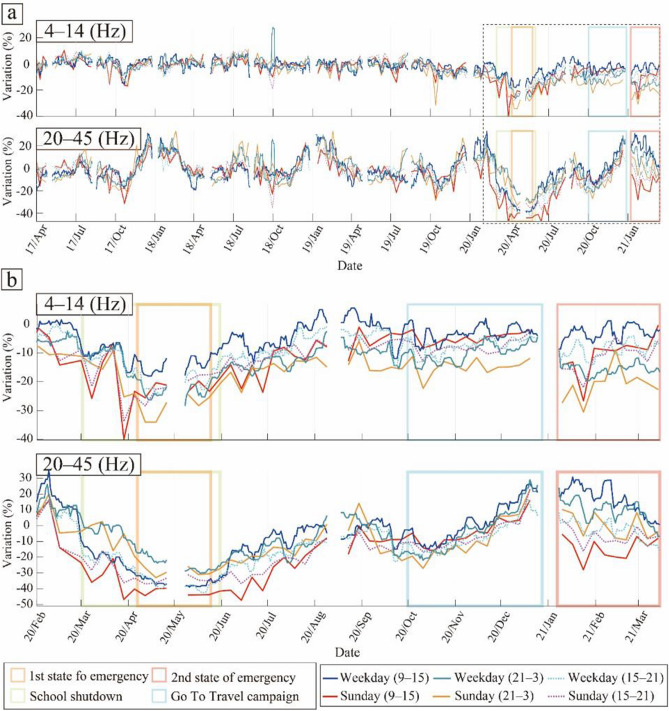
Temporal variations of PSD depending on the day of the week. (**a**) From April 2017 to March 2020. (**b**) Enlarged view of the black dashed square in (**a**). Each solid line indicates the PSD under each condition shown in the right bottom box. The orange and pink squares represent the periods of first and second states of emergency in Tokyo, respectively; the green square represents the period of school closures; and the light blue square represents the period of the Go To Travel campaign.

Figure 5 shows the results of the PSD considering both the day of the week and time of day. The temporal variations of PSD with the day of the week showed strong seasonal variations in the high-frequency range (20–45 Hz), whereas they were not clear in the low-frequency range (4–14 Hz) (Fig. 5a). After the outbreak of COVID-19, the differences in the temporal variations of PSD by day of the week became more pronounced (Fig. 5b). The difference in the daytime PSD between weekdays and Sundays was significant after the outbreak of COVID-19 (blue and red solid lines in Fig. 5b).

In the low-frequency range (4–14 Hz), the nighttime PSD decreased the most after the outbreak of COVID-19 in most periods, regardless of the day of the week, similar to the results for each time period (Figs. 4b and 5b).

In the high-frequency range (20–45 Hz), the PSDs decreased significantly during the period of school closures and the first declared state of emergency, whereas the variations differed for each day of the week in the following periods. The PSD recovered rapidly on weekdays after the state of emergency was lifted, while it recovered only gradually on Sundays. Although the PSD variations depending on the time of day also showed gradual recoveries after the first state of emergency (Fig. 4b), the trend was most influenced by the gradual recovery on Sundays. During the Go To Travel campaign, the differences based on the day of the week became quite small in the high-frequency range. During the second state of emergency, the variations of PSD were different on different days of the week. The daytime PSD on Sundays showed a significant reduction, whereas that on weekdays did not change.

### Seasonal effects

The PSDs varied significantly depending on the restrictions of human activities or economic measures during the COVID-19 pandemic, but also showed clear seasonal variations (Figs. 3, 4, 5). In particular, in the frequency range of 20–45 Hz, the seasonal variations were as large as those related to COVID-19. To identify stations where the seasonal variations have a strong annual cycle, we calculated the correlation coefficient ($CC$) for annual temporal changes in relative noise levels between April 2017 to March 2018 and Aril 2018 to March 2019 using the following equation:

2 $$CC=\frac{cov\left({y}_{2017},{y}_{2018}\right)}{{\sigma }_{{y}_{2017}}{\sigma }_{{y}_{2018}}},$$

where ${y}_{2017}$ and ${y}_{2018}$ represent the smoothed annual changes in noise level with a 30-day moving average for April 2017 to March 2018 and Aril 2018 to March 2019, respectively; and ${\sigma }_{{y}_{2017}}$ and ${\sigma }_{{y}_{2018}}$ represent the variances of ${y}_{2017}$ and ${y}_{2018}$, respectively.

Using maps of the correlation coefficients estimated at each station (Fig. 2b,c), we identified stations with strong and consistent seasonal effects. In the frequency range of 4–14 Hz, the number of stations with high correlation coefficients was limited. In the frequency range of 20–45 Hz, however, many stations yielded high correlation coefficients, while some areas had low correlation coefficients. For instance, around Tokyo station (yellow star in Fig. 2a), we observed a low correlation for weekdays in the daytime for the frequency range of 20–45 Hz. Low correlations can be attributed to weak seasonal effects and non-periodic changes in noise levels related to irregular local events and changes in the environment.

**Figure 6:**
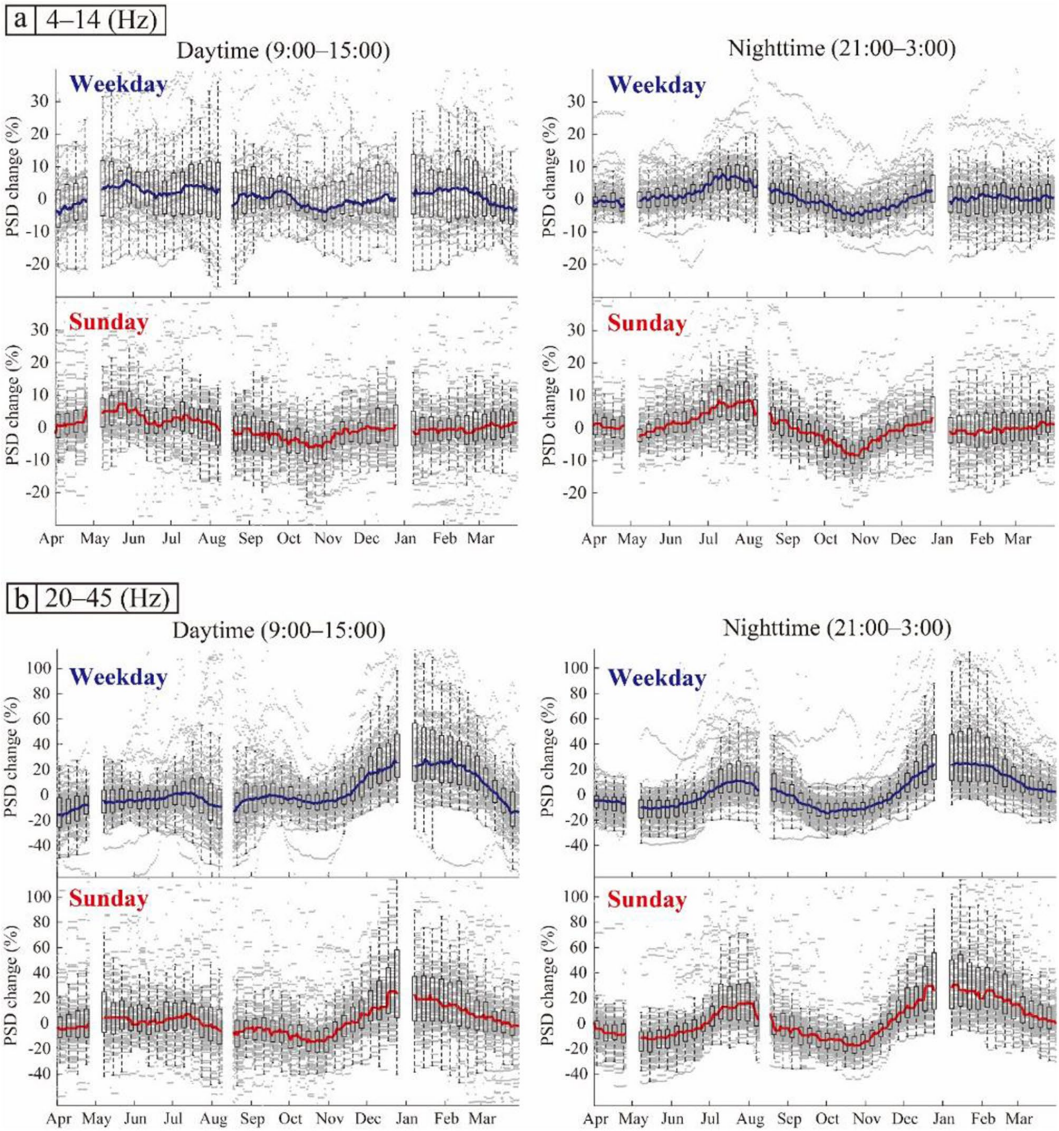
Seasonal variations in the relative noise level in the frequency range (**a**) 4–14 Hz and (**b**) 20–45 Hz. Left and right panels show the result of daytime and nighttime, respectively. Gray dots represent the seasonal variations at each station which show correlation coefficients > 0.2 (circles in Fig. 2b,c). The blue and red solid lines indicate the median values on weekdays and Sunday, respectively. The boxes represent the first and third quartiles and the error bars represent the minimum and maximum values within the ×1.5 the interquartile range from the first and third quartiles. The boxes and error bars were plotted for every 7 days.

Annual seasonal change was defined as the mean value of changes from April 2017 to March 2018 and from April 2018 to March 2019. The annual seasonal changes showed significant variations depending on the station (gray dots in Fig. 6). To visualize the overall trend of the variations, the median value of the annual seasonal changes (solid lines in Fig. 6) at the stations, which showed correlation coefficients > 0.2 (circles in Fig. 2b,c), was used. To stabilize the seasonal variation, we ignored stations that showed correlation coefficients < 0.2. The median values showed a clear seasonal variation on each day of the week and at each time point.

In the low-frequency range (4–14 Hz), the nighttime seasonal changes showed a strong PSD from July to August and a weak PSD from October to November (Fig. 6a). The daytime seasonal changes did not show a clear trend, and the error bars were much larger than the seasonal changes (boxes and dashed lines in Fig. 6a).

In the high-frequency range (20–45 Hz), the daytime seasonal changes for the weekdays showed a clear trend (Fig. 6b) with a large peak (from late November to early March) and two small dips (around late March and August). The two small dips coincided with long school holidays (roughly from March 25 to April 5 and from July 20 to August 31 at elementary schools in Tokyo). Therefore, we concluded that this noise reduction was mainly related to school activities. The nighttime seasonal changes showed a strong seasonal variation on both weekdays and Sundays (right panels in Fig. 6b), increasing during the summer and winter. An increase in PSD in summer was observed at night in both frequency bands.

### Temporal changes in relative noise level during the COVID-19 pandemic

**Figure 7:**
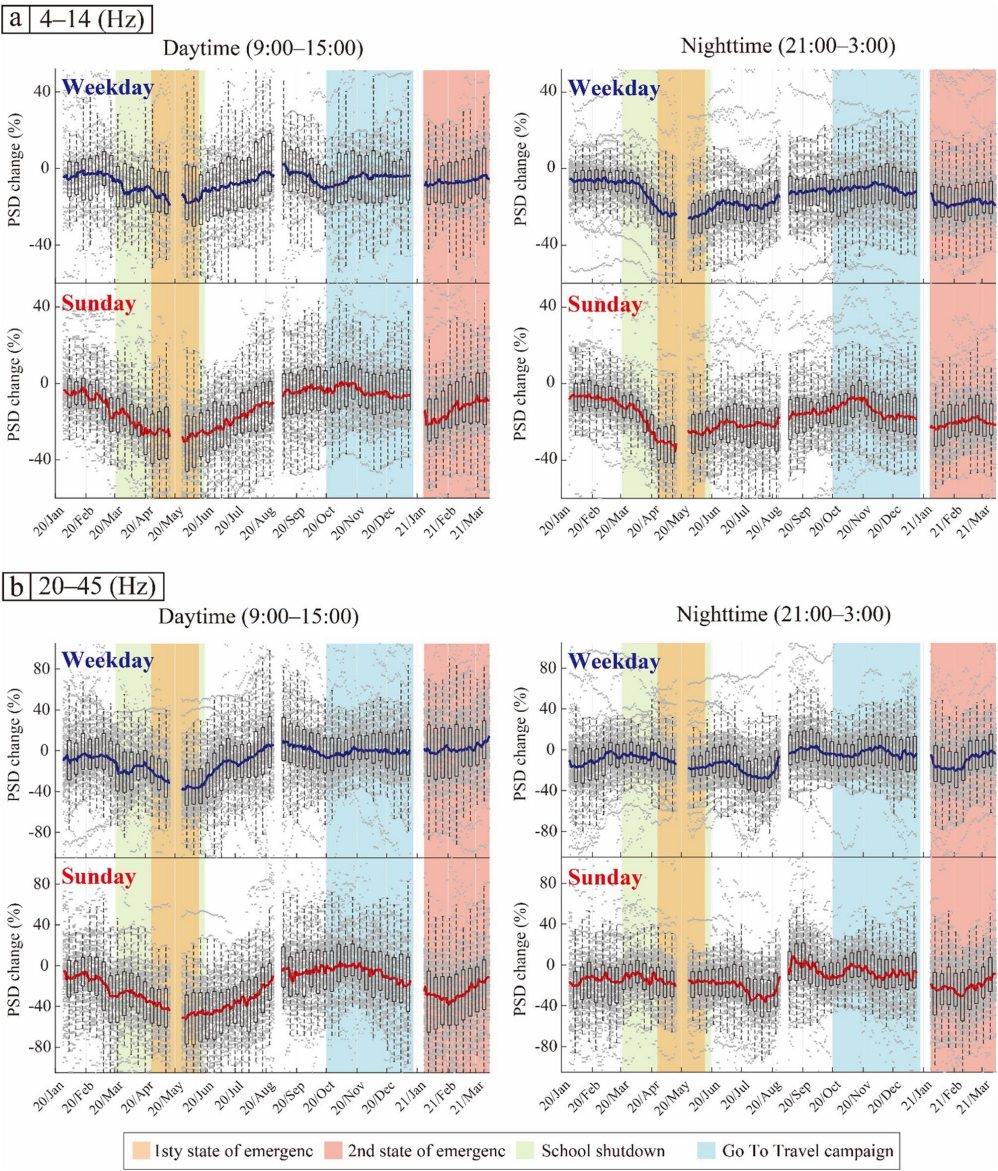
Corrected temporal changes in the relative noise level in the frequency range (**a**) 4–14 Hz and (**b**) 20–45 Hz. Left and right panels show the result of daytime and nighttime, respectively. The gray dots represent the corrected temporal changes at each station that show correlation coefficients > 0.2 (circles in Fig. 2b,c). The blue and red solid lines indicate the median values on weekdays and Sunday, respectively. The boxes represent the first and third quartiles and the error bars represent the minimum and maximum values within the ×1.5 the interquartile range from the first and third quartiles. The boxes and error bars were plotted for every 7 days. The orange and pink shaded areas represent the periods of the first and second states of emergency in Tokyo, respectively; the green shaded area represents the period of school closures; and the light blue shaded area represents the period of the Go To Travel campaign.

The temporal changes in the relative noise level were corrected for seasonal changes by deducting the annual seasonal changes. The corrected temporal changes in the relative noise level from January 2020 to March 2021 at each station and their median values are shown by gray dots and solid lines in Fig. 7, respectively. The median values clearly decreased around the periods of school closures and declared states of emergency in most cases (Fig. 7).

In the low-frequency range (4–14 Hz), the PSDs decreased in all cases during the first state of emergency; after that, the PSDs gradually recovered, almost reaching pre-COVID-19 pandemic noise levels, until the beginning of the Go To Travel campaign (Fig. 7a). During the Go To Travel campaign, no further increase was apparent. On Sunday nights, the PSD decreased rapidly since November. In the daytime on Sundays, the PSD decreased significantly with the second declared state of emergency.

In the high-frequency range (20–45 Hz), the daytime PSD decreased significantly around the periods of school closures and declared states of emergency (Fig. 7b). The daytime PSD on weekdays recovered rapidly after the first state of emergency was lifted, while that on Sundays did not immediately recover, similar to the results for the day of the week (Fig. 5b).

During the Go To Travel campaign, the PSD did not change clearly in all cases, although the results for the days of the week showed a significant increase in PSD during this period (Figs. 5b and 7b). Thus, correcting for the annual seasonal variations revealed that most stations recorded PSD variations similar to those pre-COVID-19 pandemic. After the declaration of the second state of emergency, the PSDs clearly decreased, except for the daytime on weekdays. These trends are similar to the results shown in Fig. 5b. Around the end of the second state of emergency, the PSDs increased, although the results for the days of the week showed a decrease (Figs. 5b and 7b). At night, the PSDs clearly decreased from July to August (right panels in Fig. 7b). This period corresponds to a period of large seasonal variation of PSD in summer (Fig. 6b).

## Discussion and conclusions

**Figure 8:**
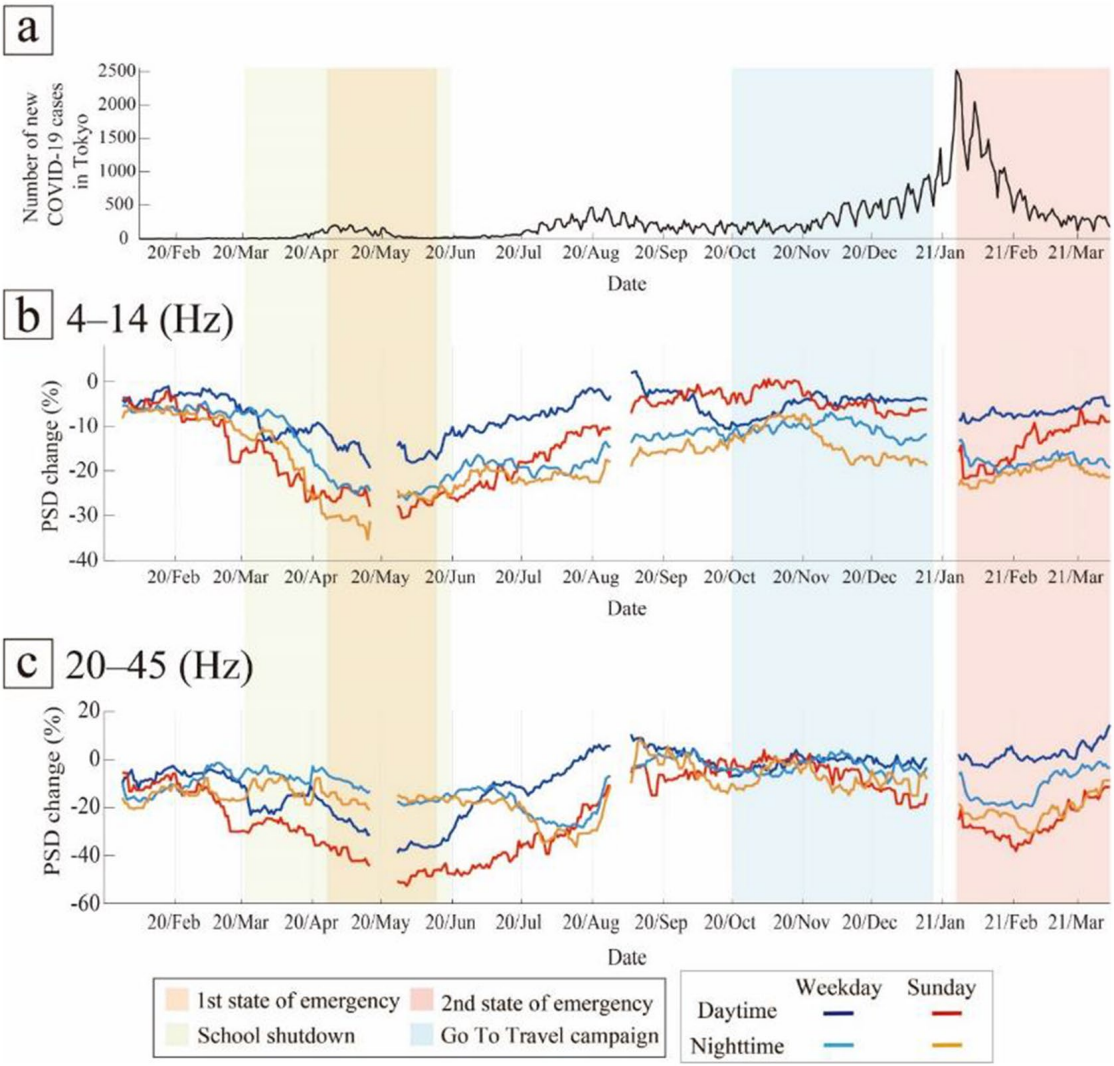
(**a**) Number of new COVID-19 cases in Tokyo (same as Fig. 1). (**b**, **c**) Comparison between temporal changes in the noise level in the frequency ranges (**b**) 4–14 Hz and (**c**) 20–45 Hz. The orange and pink shaded areas represent the periods of the first and second states of emergency in Tokyo, respectively; the green shaded area represents the period of school closures; and the light blue shaded area represents the period of the Go To Travel campaign.

Using continuous seismic data recorded by MeSO-net stations, we monitored the changes in the seismic noise levels around Tokyo from April 2017 to March 2021. To constrain the effect of human activities on anthropogenic seismic noise, we studied the temporal variations of PSD depending on the time of day and day of the week (Figs. 4 and 5). The PSDs showed significant variations related to restrictions and measures during the COVID-19 pandemic. Furthermore, the PSDs, especially in the frequency range of 20–45 Hz, showed clear seasonal or periodic variations (Figs. 4a and 5a). To confirm this seasonal effect, we estimated seasonal changes and corrected for these changes in the noise level using stations where the correlation of the PSDs of the periods April 2017 to March 2018 and April 2018 to March 2019 yielded correlation coefficients > 0.2. Although the number of stations with correlation coefficients > 0.2 was limited, especially in the frequency range of 4–14 Hz (Fig. 2b,c), the median value of the seasonal variations showed significant features in both frequency ranges (Fig. 6). Most seasonal changes showed a strong PSD and a weak PSD season. Since the seasonal changes were as large as the changes related to COVID-19, we corrected for the temporal changes in the relative noise level by deducting the annual seasonal changes. Most of the corrected temporal variations of the PSDs showed the largest reduction during the first state of emergency for both time of day and day of the week (Figs. 4, 5, and 7). To compare the features of the corrected temporal variations of the PSDs, we plotted their median values on the same graph (Fig. 8).

During the first state of emergency, the PSD decreased the most under all conditions except for at night in the high-frequency range (orange shaded area in Fig. 8). This was because the first state of emergency had tighter restrictions than the second state of emergency. For instance, school closures and forgoing unnecessary outings were requested in the first state of emergency, whereas during the second state of emergency, school activities and leisure events continued. In the latter period, the nighttime PSD in the high-frequency range did not show a reduction (light blue and orange lines in Fig. 8c). Moreover, the results for the time of day in this range also showed only a small reduction in the first state of emergency (gray and right orange lines in Fig. 3b). The first state of emergency was less sensitive to the noise level at night (21:00–3:00).

In addition, the daytime PSD on weekdays in the high-frequency range further decreased after the beginning of the school closures, but the decrease was smaller (blue line in Fig. 8c). During the first state of emergency, in addition to the school closures, the majority of educational and recreational facilities were ordered to close; therefore, noise decreased more significantly during this period than during the school closure period. The daytime results for weekdays during the first state of emergency were similar to the two-step PSD reduction reported by Yabe et al.^16^, and these trends were also confirmed in the wider Tokyo area that was covered by the stations used in this study.

After the first state of emergency was lifted, the daytime PSD immediately recovered on weekdays (blue lines in Fig. 8b,c). In contrast, the recovery on Sundays was gradual (red lines in Fig. 8b,c). The PSDs continued to recover until the period of the Go To Travel campaign. The weekday noise level strongly reflected the effects of the lifting of the state of emergency, whereas the Sunday noise level likely reflected the concern of people about COVID-19, as most people continued to avoid non-essential outings even after the state of emergency was lifted.

The nighttime PSD in the high-frequency range showed a significant reduction from late June to late August (light blue and orange lines in Fig. 8c). In this period, the seasonal variation of PSD at night increased (right panels in Fig. 6b). Therefore, this reduction could indicate a lack of summer night activities, as in previous years.

During the Go To Travel campaign (light blue shaded area in Fig. 8), the PSDs showed no clear variation except for at night on Sundays in the low-frequency range, even though the results for time of day and day of the week in the high-frequency range showed significant increases during this period (Figs. 4b and 5b). Thus, the increases in PSD in the high-frequency range during this period were not due to the Go To Travel campaign, but rather to seasonal changes. The rate of variation of PSD recovered was almost zero, and the difference between each condition became small during this period, especially in the high-frequency range (Fig. 8c). We assume that the level of social activities was close to the normal conditions prior to the COVID-19 pandemic. Before the COVID-19 pandemic, the differences in each condition were smaller than those after the outbreak of COVID-19 (Figs. 4a and 5a).

Following the declaration of the second state of emergency, all PSDs except for the daytime PSD on weekdays showed a significant reduction in both frequency ranges (pink shaded area in Fig. 8). The second state of emergency was focused on restricting activity at night. Accordingly, the nighttime PSD followed the effect of the second state of emergency. Furthermore, if the Sunday noise levels indeed strongly reflect the concern of the people about COVID-19, most people avoided non-essential outings voluntarily. The PSDs gradually increased from the middle of the second state of emergency, suggesting a gradual reduction in public concern about COVID-19 following a decrease in the number of infections.

In this study, we analyzed the differences in the temporal variations of PSD in two different frequency ranges, for time of day, and day of the week during the COVID-19 pandemic. Our results will help identify specific sources of anthropogenic seismic noise. Furthermore, recently, because of the development of permanent high-quality seismic networks and seismic processing techniques, it has become possible to use specific anthropogenic noises to visualize near-surface structures. Anthropogenic seismic noises, such as traffic and trains, were used to extract shear, compressional, or surface waves propagating between pairs of stations by computing cross-correlations for visualization or monitoring^21, 22^. Identifying dominant anthropogenic noise is thus useful in geophysical exploration using passive seismic noise. Studying anthropogenic seismic noise is an efficient way to characterize and monitor specific human activities and visualize underground structures.
